# Possible Involvement of Vitamin C in Periodontal Disease-Diabetes Mellitus Association

**DOI:** 10.3390/nu12020553

**Published:** 2020-02-20

**Authors:** Maria Bogdan, Andreea Daniela Meca, Mihail Virgil Boldeanu, Dorin Nicolae Gheorghe, Adina Turcu-Stiolica, Mihaela-Simona Subtirelu, Lidia Boldeanu, Mihaela Blaj, Gina Eosefina Botnariu, Cristiana Elena Vlad, Liliana Georgeta Foia, Petra Surlin

**Affiliations:** 1 Department of Pharmacology, University of Medicine and Pharmacy, 200349 Craiova, Romania; bogdanfmaria81@yahoo.com (M.B.); andreea_mdc@yahoo.com (A.D.M.); 2 Department of Immunology, University of Medicine and Pharmacy, 200349 Craiova, Romania; 3 Department of Periodontology, University of Medicine and Pharmacy, 200349 Craiova, Romania; dorinngheorghe@gmail.com (D.N.G.); surlinpetra@gmail.com (P.S.); 4 Department of Pharmacoeconomics, University of Medicine and Pharmacy, 200349 Craiova, Romania; adina.turcu@gmail.com (A.T.-S.); mihaela.subtirelu@yahoo.com (M.-S.S.); 5 Department of Microbiology, University of Medicine and Pharmacy, 200349 Craiova, Romania; barulidia@yahoo.com; 6 Department of Surgery, University of Medicine and Pharmacy “Gr. T. Popa”, 700115 Iasi, Romania; 7 Department of Internal Medicine, University of Medicine and Pharmacy “Gr. T. Popa”, 700115 Iasi, Romania; ginabotnariu66@gmail.com (G.E.B.); vladcristiana@gmail.com (C.E.V.); 8 Department of Biochemistry, University of Medicine and Pharmacy “Gr. T. Popa”, 700115 Iasi, Romania; lilifoia@yahoo.co.uk

**Keywords:** vitamin C, ascorbic acid, diabetes mellitus, periodontal disease

## Abstract

Ascorbic acid (vitamin C) is an important water-soluble vitamin found in many fruits and vegetables. It has well-documented beneficial effects on the human body and is used as a supplement, alone or in combination with other vitamins and minerals. Over recent years, research has focused on possible new therapeutic actions in chronic conditions including periodontal disease (PD). We conducted a systematic review on clinical trials from four databases (PubMed, Clinical Trials, Cochrane, Web of Science) which measured plasmatic/salivary levels of ascorbic acid in PD–diabetes mellitus (DM) association. Six studies were included in our review, three of them analyzing patients with different grades of PD and DM who received vitamin C as a treatment (500 mg vitamin C/day for 2 months and 450 mg/day for 2 weeks) or as part of their alimentation (guava fruits), in combination with standard therapies and procedures. Decreased levels of vitamin C were observed in PD patients with DM but data about efficacy of vitamin C administration are inconclusive. Given the important bidirectional relationship between PD and DM, there is a strong need for more research to assess the positive effects of ascorbic acid supplementation in individuals suffering from both diseases and also its proper regimen for these patients.

## 1. Introduction

### 1.1. Diabetes Mellitus, Periodontal Disease and Their Interaction

Diabetes mellitus (DM) is a chronic metabolic disease that alters the physiologic circuit of glucose. If left untreated it can lead to deadly complications such as cardiovascular disease, brain stroke, loss of sight or renal insufficiency. In order to enter a cell and to be used for metabolic purposes, glucose requires the presence of insulin. The pancreas produces insulin, but in some situations, it does not produce enough [1]. This is the cause of type I diabetes, also known as “insulin-dependent DM” because patients need insulin administration during treatment. In other situations, the cells may be resistant and insensitive to insulin action, therefore preventing glucose metabolism. This happens in type II diabetes, which is often associated with obesity. The first stages of type II diabetes treatment include diet control, exercise, and antidiabetic medication but may eventually lead to insulin administration as well. Type II DM is the most common type of adult diabetes, being diagnosed in about 90% of diabetes cases [1]. 

Periodontal disease (PD) is a chronic inflammatory disorder and a worldwide public health challenge. Since the 1960s scientific evidence has been published regarding an association between DM and periodontitis [2]. It is related to many other chronic diseases, such as cardiovascular disease, inflammatory bowel disease, rheumatoid arthritis, respiratory tract infection and Alzheimer’s disease, displaying a particular interest in the relationship between oral and systemic health [3,4,5]. PD is caused by specific oral microorganisms, such as *Porphyromonas gingivalis, Treponema denticola*, *Tannerella forsythia* and *Aggregatibacter actinomycetemcomitans* [6,7,8], inducing loss of periodontal ligament and alveolar bone, also representing the primary cause of tooth loss [9]. Periodontal impairment is influenced by many risk factors, including alcohol, stress, smoking, heredity, DM and endocrinological changes (pregnancy or menopause); thus, maintaining periodontal health becomes a real challenge [10]. The human oral microbiome is important in the pathogenesis of PD, nutrition being a significant aspect in promoting periodontal homeostasis through antioxidant and immunomodulatory effects on bone metabolism [10,11,12].

Since 2012, the American Diabetes Association has been including the periodontal examination of diabetic patients in its “Standards of Medical Care for Diabetes”. This action has been motivated by the fact that PD was officially recognized as a complication of DM, together with the five other vascular-derived ones (retinopathy, neuropathy, etc.) [13].

Conversely, DM is also credited with an important influence on the pathogenesis process of certain types of PD, as illustrated by the latest classification of periodontal conditions, issued by the European Federation of Periodontology and the American Academy of Periodontology in 2018 [14]. The bidirectional relationship between the two disorders is currently well documented, opening perspectives of common management of diabetic and periodontal patients, in terms of prevention, early diagnosis and integrated treatment protocols [15].

The negative impact that diabetic pathology has on the periodontal status of affected patients has been explained by various mechanisms. From a cellular perspective, it seems that the mobility, activity and efficiency of immune cells, such as polymorphonuclear leukocytes is decreased in a diabetic setting, favoring the aggressive actions of periodontal bacterial pathogens [16,17]. Also, the antibacterial capacity of the saliva and gingival crevicular fluid (GCF) could be downregulated in DM patients, further enhancing the growth of harmful bacteria. In addition to this, periodontal ligament fibroblasts have been shown to decrease chemotaxis when placed in vitro in a hyperglycemic environment [18]. The glucose-rich GCF of DM patients is one such environment, which may explain the difficult periodontal wound healing and the reduced local host response to bacterial attack, all favoring the onset of periodontal inflammation and damage.

Proinflammatory markers, which drive the inflammatory reaction, are secreted by certain immune cells when they are stimulated by bacterial antigens. It has been shown that the immune cells of DM patients over-react to bacterial antigen stimulation, causing an overproduction of proinflammatory markers. Consequently, a more intense inflammatory periodontal reaction is triggered in DM patients, causing the rapid destruction of periodontal tissues [19,20]. The involved proinflammatory markers include major cytokines, such as interleukin 1β (IL-1β), tumor necrosis factor-alpha (TNF-α) and prostaglandin E2 (PGE2), which are all majorly upregulated in DM patients’ GCF compared to non-DM patients. When compared, the GCF levels of PGE2 and IL–1β were higher in DM patients’ samples than in non-DM ones, in similar settings of PD inflammation and dissolution.

Poorly controlled diabetes is a key factor in the onset of aggressive and destructive forms of PD [21].

Some studies support the direct connection between high PD prevalence and severity in DM patients [22]. This seems to be especially true for type 2 DM patients, who are more prone to difficulty in glycemic control [23]. Poor glycemic control can also impact the outcome of periodontal treatment. Patients with well-controlled glycemia have been shown to reach similar results after nonsurgical periodontal treatment (scaling and root planning) as those of non-DM patients at a four month recall [24]. In contrast, a less favorable response to treatment can be expected from DM patients with uncontrolled glycemia [25]. Periodontal surgery also delivers similar results in terms of periodontal pocket reduction for well-controlled glycemia DM patients compared to non-diabetic ones [26]. Therefore, favorable results can be expected when treating periodontally compromised DM patients with stable glycemia levels.

It was also found that PD patients with undiagnosed DM exhibit significantly increased glycosylated hemoglobin (HbA1C) serum levels compared to periodontally healthy individuals, and PD was positively correlated with serum levels of (HbA1C) before DM onset [27]. If PD acts as an aggravating cofactor for later DM complications, its treatment may be a way to improve the diabetic status and to stabilize glycemic levels, thereby preventing the onset of dangerous complications.

### 1.2. Oxidative Stress and Reactive Oxygen Species—Background

Oxidative stress is a state of imbalance between oxidants and antioxidants in favor of oxidants, leading to harmful effects [28]. Oxidants, also called reactive oxygen species (ROS), include free radicals such as O_2_• - (superoxide), ONOO ^-^ (peroxynitrite) and HO• (hydroxyl) and nonradicals, such as H_2_O_2_ (hydrogen peroxide), are products of aerobic cell metabolism by reducing oxygen molecules [29]. There are many sources of ROS, mainly generated by enzymes such as xanthine oxidase, cyclooxygenase, lipooxygenase, myeloperoxidase, cytochrome P450 monooxygenase, uncoupled nitric oxide synthase (NOS), peroxidase and nicotinamide adenine dinucleotide phosphate (NADPH) oxidase. They arise intracellularly, extracellularly, or in specific intracellular compartments [30] and are generated by polymorphonuclear lymphocytes through NADPH oxidase [31].

Oxygen-derived free radicals are oxidative agents produced during events such as mitochondrial respiration and phagocytosis, causing post-translational modifications of proteins, with an impact on cell signaling, gene expression and other physiological processes [32]. Low concentrations of ROS enhance antioxidant response by activating a nuclear factor erythroid 2-related factor 2, promoting cell survival [33]. ROS-induced impairment of glycocalyx, cell membranes and junctions contribute to increased permeability and leukocyte and thrombocyte adhesion, with subsequent local activation of inflammation and coagulation, leading to loss of endothelial vasodilation potential and attenuation of vasoconstrictor response [34].

**Figure 1:**
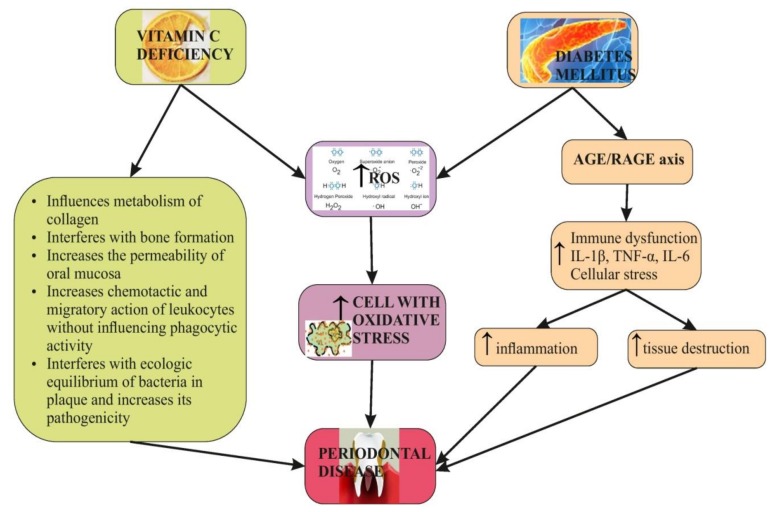
The interrelation between vitamin C, diabetes mellitus (DM) and periodontal disease (PD).

There is also a documented link between oxidative stress, DM and PD, with the oxidative-stress-mediated changes in the inflammatory pathways being possible mechanisms in affecting periodontal tissues [35] (Figure 1). DM and PD involve significant impairment of immune system regulation, while hyperglycemia contributes to advanced glycation end products (AGE) formation and extended levels of proinflammatory cytokines IL-1β, interleukin 6 (IL-6) and TNF-α [36] (Figure 1).

### 1.3. Vitamin C, DM, and PD

In the 1920s, the forthcoming Nobel laureate Albert Szent-Györgyi from Szeged University, Hungary, identified vitamin C and its role in preventing and treating scurvy [37]. Ascorbic acid (vitamin C) is an essential water-soluble vitamin that cannot be synthesized by the human organism [38,39]. It demands a regular and appropriate intake from natural sources, like citrus fruits, mango, strawberries, kiwi, papaya, green leafy vegetables, tomatoes and broccoli [37], to hamper hypovitaminosis C that is relatively common in Western populations [38,39].

Synthetic vitamin C derived from chemicals is similar to that contained in fruits and vegetables [40]. The main route of administration for ascorbic acid is oral ingestion from food or supplements. Healthy individuals generally need 0.1–0.2 g daily doses. Intravenous administration is used in critically ill patients requiring high doses (1–4 g/day) of this nutrient [41,42]. It is quickly eliminated by the kidneys with a half-life of approximately two hours [41,43].

L-ascorbate, the reduced form of vitamin C, is a physiological antioxidant [44]. Antioxidants are molecules that can donate a hydrogen atom or an electron to a radical, ceasing chain reactions [45] such as metal chelation and protecting cells from radiation damage and the formation of nonradical and nonreactive end products of antioxidant enzymes [28].

Vitamin C improves immune function and facilitates iron absorption, reduction of folic acid derivatives and synthesis of collagen, cortisol, catecholamines and carnitine [46]. Vitamin C also improves the synthesis of prostaglandins PGE1 and PGI2 and nitric oxide (eNO); it has a cytoprotection role, antimutagenic activity, vasodilatory action and inhibitory effect on platelet aggregation, being useful in type 2 DM and high blood pressure [47].

Ascorbic acid deficiency has been associated with stroke, DM, cancer, cardiovascular disease, infectious diseases and sepsis [41].

In type 2 DM the plasma levels of IL-6 and TNF-α are elevated, lipid peroxides are increased and unsaturated fatty acids, especially arachidonic acid (AA) and lipoxin A4 (LXA4), are reduced [48]. Besides, the usefulness of vitamin C in the management of type 2 DM is confirmed by a study conducted by Mason et al. [49] which recorded that oral vitamin C (1000 mg daily) reduced hyperglycemia. This action arose after a decrease of plasma isoprostane-F2 in these subjects and indicated that the beneficial action of vitamin C was not only due to its antioxidant property but also to its ability to improve PGE1, PGI2, LXA4 and eNO [49].

Oxidative stress plays an important role in the development of vascular complications in type 2 DM, and the increase of ROS level is due to decreased production of some enzymatic/nonenzymatic antioxidants, i.e., catalase, superoxide dismutase (SOD) and glutathione peroxidase (GSH-Px), leading to the development of diabetic complications [50]. Free radical formation in type 2 DM is accomplished by nonenzymatic proteins glycation, oxidation of glucose, increased lipid peroxidation, inducing enzyme damage and increased insulin resistance [51]. Insulin signaling is modulated by ROS/RNS (reactive nitrogen species) in two ways: first, in response to insulin, ROS/RNS exerts a physiological function; second, the ROS and RNS pathway negatively regulates insulin signaling, contributing to development of insulin resistance, which is a risk factor for type 2 DM [52].

Oxidative stress and ROS induce complications of DM including coronary heart disease, neuropathy, nephropathy, retinopathy and stroke [50]. Hyperglycemia plays a role in the generation of oxidative stress leading to vascular endothelial dysfunction of patients with DM and, together with dyslipidemia, develops macroangiopathies, which cause oxidative stress leading to atherosclerosis [50]. Vitamin C acts as an antioxidant by detoxification of ROS, hence being an important biomarker of oxidative stress, but, depending on the situation, it might promote toxicity via pro-oxidant formation [51].

Vitamin C plays a key role in maintaining the integrity of the connective tissues, thus of the periodontium. It is a powerful antioxidant, particularly at the intracellular level, being an enzymatic cofactor in metabolic reactions (hydroxylation of proline and lysine needed to stabilize collagen structures during its synthesis) [10].

Regarding the interplay between vitamin C and PD, the results of observational studies are contradictory, depending on the parameter evaluated, with several studies reporting no association between vitamin C and PD [10]. However, Nishida et al. [53] in their study with 12,419 participants identified a dose-dependent relationship between the vitamin C intake and the number of people with PD. Contrary to these results, a relation between vitamin C deficiency and PD was not recorded by other researches [54,55].

Vitamin C intake is necessary to avoid periodontal issues, but when a pathological condition has been established, a supplement with vitamin C is not sufficient to cure periodontal pathology [10]. Also, the effect of vitamin C combined with chlorhexidine can prevent and slow down the PD progression [56].

Monea et al. [57], in a case-control study (which included 10 patients with type 2 DM and 8 healthy adults), observed significantly increased malondialdehyde (MDA) levels in periodontal tissues, suggesting increased lipid peroxidation and decreased glutathione tissue levels (GSH), resulting a change of the local defense mechanism. Thus, histological aspects in the periodontal tissues of diabetic subjects confirm the involvement of oxidative stress [57].

In a study with murine models with diabetes, Li et al. [58] found that simultaneous periodontitis and DM synergistically aggravated both local and systemic oxidative lesions, being correlated with more severe periodontal destruction in diabetic periodontitis.

In a study with 10,930 patients, Lee et al. [59] found that in patients with DM between the ages of 30 and 49, there was a significant link between vitamin C intake and periodontitis. In the stratified analysis, the aforementioned association was highlighted among patients with type 2 DM. When there was inadequate vitamin C intake, diabetic subjects were more sensitive to oxidative stress, developing PD. These results pointed out the crucial role of vitamin C in promoting periodontal health among adults [59].

In a prospective cohort study that included 579 men, Dietrich et al. [60] observed that participants with periodontitis had a lower intake of vitamin C, increased risk of DM, higher levels of bleeding, bacterial plaque, loss of attachment and fewer teeth.

Considering the positive association between PD and ischemic heart disease, serum and salivary levels of vitamin C were analyzed in patients suffering from both conditions and were found to be lower compared to PD patients and healthy individuals [61].

The present study aims to systematically review the available clinical data about the plasmatic and salivary levels of ascorbic acid in patients affected by PD and DM and about possible beneficial effects of ascorbic acid supplementation in PD–DM association.

## 2. Methods

The protocol of the review was developed following the Preferred Reporting Items for Systematic Reviews and Meta-Analyses (PRISMA) statement guidelines [62] and was designed to gather the results of clinical trials in patients with different grades of PD and DM whose plasma levels of vitamin C were determined.

### 2.1. Study Selection Criteria

The population of interest for this review included patients with a current diagnosis of both chronic PD and DM (type 1 or type 2). All the studies which met the following inclusion criteria were included in this systematic review: (1) written in English; (2) published before 8 September 2019; (3) investigating association between vitamin C, PD and DM; (4) clinical trials conducted on adults; and (5) using quantitative methods of data collection. The design of the targeted studies, which were of interest, depended on the dosage and frequency of administration of ascorbic acid, both as therapeutic administration and as part of the patients’ usual alimentation. Other types of studies, such as cohort, randomized and cross-sectional surveys were also included. Studies that included only plasmatic or salivary measurements of vitamin C were also of interest and included. Articles’ exclusion criteria were: (1) written in a language other than English; (2) reviews and animal studies; (3) abstract only or no abstract; (4) not mentioning whether the patients had DM or not; or (5) not measuring ascorbic acid plasma/salivary levels.

### 2.2. Literature Search

The electronic literature search was conducted by two independent authors (M.B. and A.D.M.) within the following databases: PubMed, Clinical Trials, Cochrane and Web of Science.

The inclusion criteria were defined according to the PICO model: population (P = “human adults”), intervention or exposure (I = “impact of vitamin C on patients with PD and DM”), comparison (C = “dosage and frequency of dosage for vitamin C, received as treatment or as part of alimentation; different concentrations of plasmatic vitamin C”), and outcome (O = “measurement of periodontal status using specific disease parameters”). The following PICO question was used: “Is vitamin C associated with an improvement of periodontal status in patients with DM?”

Four types of searches in each database were performed with the exact term combination: type 1—“vitamin C AND periodontal disease AND diabetes mellitus” OR type 2—“ascorbic acid AND periodontal disease AND diabetes mellitus” OR type 3—“vitamin C AND periodontitis AND diabetes mellitus” OR type 4—“ascorbic acid AND periodontitis AND diabetes mellitus”.

### 2.3. Selection of Studies

Both authors assessed the eligibility of all the studies and screened them, eliminating duplicates and removing all the studies that did not respect the selection criteria after assessing the content from titles and abstracts. The reviewers shared their independently obtained data and resolved decided any disagreements by general approval. The final titles were included for further data extraction and analysis.

### 2.4. Data Extraction and Analysis

The two authors independently extracted data from the final articles into an Excel template developed by the research team. The included elements were publication year, study type design, the country where the study was run, participants’ characteristics (number, age and gender), periodontal status and type of measurement, type of intervention, along with diabetes status and type of measurement. The experimental design of the final list of studies was reported to cover the duration of the study, the administration (dosage and frequency) of vitamin C, the measurement of vitamin C and their main results.

## 3. Results

**Figure 2:**
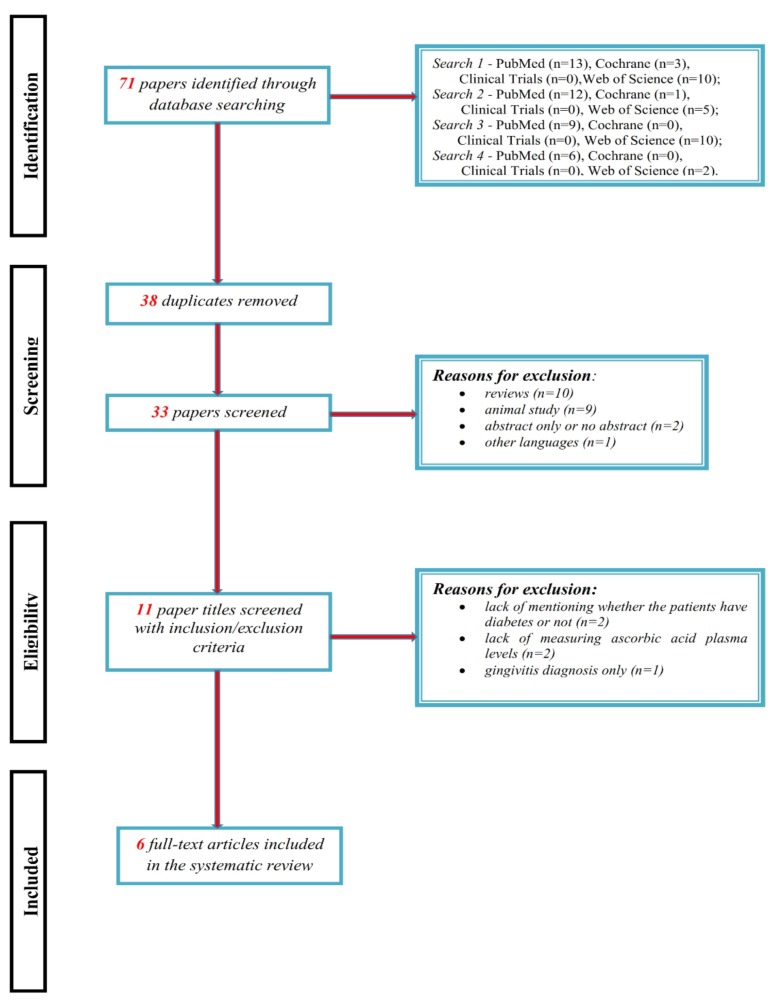
Flowchart of the systematic search based on PRISMA guidelines.

The literature search resulted in 71 articles across the four databases (PubMed, Clinical Trials, Cochrane, Web of Science), of which 33 were reviewed after duplicates (*n* = 38) were removed (Figure 2). After screening with inclusion/exclusion criteria, six papers remained for the systematic analysis. Detailed summaries of final studies are included in Table 1 and Table 2.

Two of the remaining six studies aimed to evaluate the relationship between plasma ascorbic acid levels and PD in systemically healthy and type 2 DM subjects, following the administration of vitamin C in similar 450–500 mg daily doses. Gokhale et al. [63] randomly divided participants into subgroups, using a coin-toss method: subgroup A (15 adults) receiving 450 mg chewable tablet associated with daily scaling and root planning and subgroup B (15 adults) receiving placebo as lemon-flavored sugar-free candy chewable tablets with scaling and root planning spaced over two appointments. It revealed that dietary ascorbic acid supplementation associated with scaling and root planning improved the sulcus bleeding index in subjects with gingivitis and diabetics with periodontitis. They also obtained additional results, by using Tukey’s multiple post-hoc procedures, regarding the plasma ascorbic acid levels in subgroup A, which supported their conclusion [63]. Similarly, Kunsongkeit et al. [64] assessed the administration of daily 500 mg vitamin C tablets, for 2 months in 15 adults or placebo tablets in 16 adults, both groups receiving full scaling and root planning from baseline to the last administered tablet, comparing the results within groups using Bonferroni post-hoc test. Periodontitis patients with uncontrolled type 2 DM did not exhibit evident benefits by supplementation of 500 mg/day vitamin C [64], but the differences between their results may have been generated by the recently diagnosed type 2 DM patients included in the Gokhale et al. study and uncontrolled type 2 DM patients included in the Kunsongkeit el al. study. Therefore, the progression and severity of periodontitis were greater in patients with uncontrolled diabetes and perhaps the dosage of vitamin C should have been larger to sustain the conclusions revealed in the Gokhale et al. study. Even so, both studies assumed the bidirectional relationship between periodontitis and DM, which means further assessments should be aimed by other medical specialists. 

Three studies from the ones selected [65,66,67] evaluated the effect of dietary intake of vitamin C, as an antioxidant and immunomodulatory agent, and the evolution of PD in patients with DM, without specifying certain consumed fruits or vegetables or dosage, but measuring plasmatic [65,67] or salivary concentration of ascorbic acid [66]. Thomas et al. [65] measured plasmatic ascorbic acid levels in their case-control study by using spectrophotometric quantitation on all three groups: 20 patients with type 2 DM and PD, 20 healthy patients with PD and 20 healthy patients without PD. This method was useful for comparing the micronutrient levels not only of vitamin C but also of zinc and copper in diabetic patients and healthy individuals with periodontitis, finally showing that diet plays a modifying role in the progression of periodontal disease. The idea was sustained by a statistically significant decrease in vitamin C levels in diabetic patients with periodontitis when compared to healthy individuals with periodontitis [65]. The same conclusion appeared in a cross-sectional survey, also held in India, by Patil et al. [67], even though vitamin C was measured by a different chemical method (dinitro phenyl hydrazine method). They included an additional group consisting of patients who suffered from gingivitis, an incipient form of PD, and a group of recently diagnosed patients who suffered from periodontitis and diabetes, who had not received any antidiabetic medication, before the study onset [67]. Gumus et al. [66] conducted a case-control study in Turkey by measuring the total antioxidant capacity in patients with PD, divided into three groups: 16 patients with type 1 DM, 25 patients with type 2 DM and 24 patients with no associated disease. They used the Kruskal–Wallis test, followed by the Mann–Whitney U test for the group comparisons of the salivary antioxidant levels, as well as the clinical periodontal measurements; however, their conclusion was different from the Indian studies, because vitamin C did not seem to play a major role in the pathogenesis of periodontal manifestations in diabetes. They mentioned the absence of a group with diabetes with a clinically healthy periodontium which would have enabled them to conclude whether the levels of salivary antioxidants are related to the diabetic status independently of the clinical periodontal situation [66]. This limitation of the Turkish study might have led to a different conclusion regarding the ascorbic acid levels in patients with both diabetes and periodontitis than the one commonly presented in Indian studies.

Amaliya et al. [68] organized a cohort study in Indonesia analyzing the intake of vitamin C from the dietary origin while monitoring all 98 patients using a full set of dental radiographs with long cone paralleling technique. Their results depended on the consumption of guava fruit over one month in all 53 women and 45 men, with an age range from 39 to 50 years, who were included in the study. Amaliya et al. stated that guava fruit contains 228 mg vitamin C per 100 g (USDA 2010), which implied that the consumption of one guava (without skin and kernel) results in an intake of about 400 mg vitamin C. Since the guava consumption varied between 0 and 30 guavas in the month preceding plasmatic ascorbic acid measurements, a great variation in the amount of vitamin C intake existed in their included population, possibly contributing to the significant association with alveolar bone loss. In their study, 45% of the population showed vitamin C depletion/deficiency, 70% were in a prediabetic state and 6% had untreated diabetes. Their new finding was that guava fruit consumption may play a protective role against periodontitis in the 10% malnourished population, which showed a relatively low body mass index. These conditions may have contributed to the extent and severity of alveolar bone loss in the population. It is important to mention that Amaliya et al. had a limitation because 70% of their population were in a prediabetic state and 6% had undiagnosed diabetes, which is why no relation could be assessed between HbA1c plasma levels and alveolar bone loss, probably due to the small number of subjects with HbA1c values ≥6.5% and insufficient conclusive data of the study [68].

All studies included in our systematic review had in common the assessment indication for PD, which included certain parameters: alveolar bone loss, bleeding on probing, clinical attachment level (CAL > 3 mm), the community periodontal index, pocket depth or probing pocket depths (PPDs of ≥5 mm, along with the presence of attachment loss of ≥2 mm within at least three teeth (assessed at four sites per tooth)), plaque index and the sulcus bleeding index (SBI score of ≥2).

The plaque index had the following scoring criteria: score 0—no plaque, score 1—a film of plaque adhering to the free gingival margin and the adjacent area of the tooth, seen in situ only after the application of disclosure solution or by using the probe on the tooth surface, score 2—moderate accumulation of soft deposits within the gingival pocket, or the tooth and gingival margin, which can be seen with the naked eye, score 3—abundance of soft matter within the gingival pocket and/or on the tooth and gingival margin [63]. The assessment of gingival bleeding is done on a scale of 0–5 according to the following criteria: score 0—healthy appearance of the gingiva and no bleeding upon sulcus probing, score 1—apparently healthy gingiva showing no color or contour changes and no swelling, but sulcus bleeding on probing, score 2—bleeding on probing and color change caused by inflammation, but absent swelling, score 3—bleeding on probing, change in color and slight edematous swelling, score 4—bleeding on probing, obvious color change and swelling, and score 5—spontaneous bleeding on probing, color change, marked swelling with or without ulceration [63].

The parameters used to sustain the diagnosis of DM included body mass index (BMI), glycosylated hemoglobin (HbA1C > 7%), fasting blood sugar (FBS ≥ 126 mg/dL), two-hour postprandial glucose (PPG ≥ 200 mg/dL) and random blood sugar (RBS ≥ 200 mg/dL with symptoms such as polyuria, polydipsia and polyphagia).

## 4. Discussions

Three of our selected studies [63,65,67] were held in India, with a similar number of participants (60–120) and similarly composed groups of study, classified by periodontal status, but with the first two not mentioning their age. All the Indian studies used plasmatic measurement of vitamin C and they also had in common a finding regarding the lower plasmatic levels of ascorbic acid in periodontitis and diabetic patients than periodontitis nondiabetic patients. Four of our six eligible studies [63,64,65,67] excluded patients with any systemic disorder (other than the groups with type 1 or type 2 DM), those with presence of any disease that may alter the immune system (bacterial or viral infections, hypercholesterolemia, cardiovascular events), those who had been treated with any dietary supplements, antibiotics, and anti-inflammatory drugs in the previous 6 months, those with history of smoking or tobacco consumption, those with history of using any mechanical or chemical aids for plaque control (mouthwashes) and pregnant subjects. On the other hand, two studies included smokers (for example, Gumus et al. [66] recorded patients’ smoking history), one of them targeting the smoking population to shape a conclusion (Amaliya et al. [68] included 26 heavy smokers of which the number of cigarettes per day ranged from 15 to 24 and 19 light smokers who smoked on average 8 cigarettes per day), but fulfilled the other exclusion criteria present in all the mentioned studies.

An interesting conclusion was presented in Amaliya et al.’s [68] study, referring to vitamin C as an important prophylactic and protective measure, especially in malnourished people with both DM and PD, even though they included heavy smokers with high exposure to ROS. Their results were based on the intake of about 400 mg vitamin C daily, for at least one month, by consuming approximately 30 guava fruits, a specific diet for people living in Purbasari Tea Estate in West Java, Indonesia [68]. On the contrary, Gumus et al. only supported a difference between diabetic patients regarding the evolution of PD by mentioning that subjects with type 2 DM had fewer teeth and more sites with probing depths >4 mm than patients with type 1 DM. In this study, vitamin C and the total antioxidant capacity did not appear to play a significant role in the pathogenesis of PD–DM [66].

Individuals afflicted with DM and PD may also exhibit a decrease in vitamin C concentration through a confounding factor, depression. Depression often complicates the management of other conditions (cancer, diabetes, myocardial infarction, severe trauma) or can occur secondary to other diseases such as inflammatory conditions, Parkinson’s disease and hypothyroidism [69].

Some articles demonstrated the association between poor vitamin C and increased depression symptoms [70,71], between DM and depression [72] and between severe PD [73] and depression. Studies have shown that depression is a consequence of inadequate levels of ascorbic acid [74]. More, vitamin C can reduce the problems associated with depression [75]. Depression prevalence is two to three times higher in patients with DM, with some cases remaining underdiagnosed [76]. Depression is also a risk factor for PD [77,78,79]. This is the reason some future research must be done to minimize the influence of this confounding factor and evaluate its strength using different questionnaires [80,81].

Several recent reviews analyzed the role of vitamin C in the pathophysiology of periodontal tissue damage [10,54,82,83].

Kaur et al. and Muniz et al. pointed out the beneficial effects of ascorbic acid as a dietary antioxidant on PD management in the context of the established link between PD and oxidative stress. They implied that, as a complementary treatment for PD, the use of an antioxidant has the potential to improve periodontal clinical parameters [82,83].

In 2018, Varela-Lopez et al. [10] performed a systematic review of human and animal studies, and they concluded that vitamin C may be useful for prevention or improvement of PD. They also emphasized the need for more research to clarify dosages and taking frequency of ascorbic acid supplementation.

In a systematic review from 2019, Tada and Miura [54] highlighted the effects of vitamin C on the prevention of incidence and the development of PD. The authors observed proof of the association between vitamin C, PD and DM which suggests a complex mechanism of action between ascorbic acid and the two disorders that requires further study.

In our analysis, decreased levels of vitamin C were observed in PD patients with DM but data about efficacy of vitamin C administration are few and inconclusive. Perhaps larger doses administered over a longer period of time are needed, especially for periodontitis patients with uncontrolled type 2 DM.

To our knowledge, this is the first systematic review to assess and summarize the current outcomes on the correlation between ascorbic acid levels and PD–DM interaction. There are limitations to the present study because of the heterogeneity of the included studies’ methodology. The findings of our review reflect different outcomes because of the different experimental designs.

## 5. Conclusions

Considering the complex and strong relation between DM and PD and the paucity of available clinical evidence for the effects of ascorbic acid in individuals affected by both conditions, further detailed studies should be performed to establish the efficacy of vitamin C for these patients. Besides the issue of the required doses, the frequency and duration of administration for ascorbic acid supplements need to be clarified.

## Figures and Tables

**Table 1:** General data of the eligible studies.

Reference	Study Type	Country	Participant Characteristics (Study Sample)			Periodontal Status	Measurement of Periodontal Status	Intervention	Diabetes Status	Measurement of Diabetes
			*No.*	*Age*	*Gender*					
Gumus, 2009 [66]	CC	Turkey	65	17–73 years old	M and F	16 patients with type 1 DM (5 M, 11F, age: 17–73), 25 patients with type 2 (11 M, 14F, age: 42–69) and 24 systematically healthy (control group, 10 M, 14 F, age: 22–60), all with PD	plaque-induced inflammatory PD, non-aggressive. PII, PB, gingival recession, CAL, BOP, recorded for 6 sites per tooth.	full-mouth periodontal clinical measurements	type 1 or type 2	FBS, HbA1C, and diabetes complications
Thomas, 2010 [65]	CC	India	60	adults	M and F	3 groups: group 1—20 patients with type 2 DM and PD, group 2—20 healthy patients with PD and group 3—20 healthy patients without PD	CAL measured with a Williams periodontal probe and BOP	examinations	type 2	RBS, FBS
Gokhale, 2013 [63]	RS	India	120	30–60 years old	M and F	4 groups of 30 patients each group 1: no PD, group 2: chronic gingivitis, group 3: chronic periodontitis, group 4: chronic periodontitis and recently diagnosed type 2 diabetes; randomized subjects of groups 2–4, were grouped to receive SRP with dietary supplementation of ascorbic acid for 2 weeks or only SRP; chronic periodontitis—PPD and assessment of gingival bleeding	PII, SBI for gingivitis, PPDs for chronic periodontitis, gingival bleeding	randomized subjects within groups 2–4, divided to receive either SRP or not	type 2	CPG, FBS, PPG
Amaliya, 2015 [68]	CH	Indonesia	98	39–50 years old	45 M and 53 F	remote populations deprived of oral health care—natural development of periodontitis; subjects of this population showed a mean of 30% ABL in their dentition, ranging from 19% to 54%	dental radiographs, ABL, periapical radiologic transparency	examinations	a small number of subjects (70% in prediabetic state and 6% having undiagnosed diabetes) with HbA1c values ≥6.5%	HbA1C
Patil, 2016 [67]	CS	India	100	adults	M and F	4 groups: 25 normal healthy controls, 25 gingivitis patients, 25 chronic periodontitis patients, 25 chronic periodontitis and type 2 diabetes	BOP, SBI, PPD	examinations	type 2	FBS, PPG
Kunsongkeit, 2019 [64]	Double-blind, placebo-controlled, clinical trial	Thailand	31	43–72 years old	9 M and 22 F	moderate chronic periodontitis, 2 groups: *n* = 15 who received periodontal therapy and vitamin C for 2 months and *n* = 16 who received periodontal therapy and placebo	PII, SBI, gingival index, PPD	full SRP and examinations	type 2 uncontrolled (FBS > 150 mg/dL, HbA1c > 7%)	FBS, HbA1C

Abbreviations (in alphabetical order): ABL—alveolar bone loss, BOP—bleeding on probing, CAL—clinical attachment level, CC—case-control study, CH—cohort study, CPG—casual plasma glucose, CS—cross-sectional survey, DM—diabetes mellitus, FBS—fasting blood sugar, LS—longitudinal study, PB—probing depth, PII—plaque index, PD—periodontal disease, PPDs—probing pocket depths, PPG—two-hour postprandial glucose, RBS—random blood sugar, ROS—reactive species of oxygen, RS—randomized study, SBI—sulcus bleeding index, SRP—scaling and root planning.

**Table 2:** Experimental design.

Reference	Duration	Experimental Design			Measurement of Vitamin C	Main Results
		*Dosage*	*Administration*	*Frequency*		
Gumus, 2009 [66]	2 and 1/2 years	none	none	none	measurement of antioxidants’ salivary concentrations in whole saliva samples	Subjects with type 2 DM had fewer teeth and more sites with probing depths (>4 mm) than patients with type 1 DM. Despite this, total antioxidant capacity and vitamin C concentrations did not seem to play a major role in the pathogenesis of periodontitis correlated with DM.
Thomas, 2010 [65]	Not mentioned	none	none	none	venous blood samples collected	Diabetic patients with periodontitis revealed a significant decrease in vitamin C levels.
Gokhale, 2013 [63]	4 months	450 mg	subgroups randomly divided using a coin-toss method: subgroup A (15) 450 mg chewable tablet and subgroup B (15) placebo chewable tablet	daily intake for 2 weeks or only SRP	plasma measurement	Plasma measured AAL were below the normal range in systemically healthy subjects with gingivitis and diabetics with periodontitis. Dietary AA supplementation associated with SRP improved the SBI in patients with gingivitis and PD–DM.
Amaliya, 2015 [68]	1 year	food products categorized as high (>60 mg), fair (31–60 mg), low (2–30 mg) or no vitamin C (<2 mg vitamin C/100 g)	Number of guava fruit servings	food frequency taken in the last month	plasma measurement, based on the values provided by the National Nutrient Database for standard reference	45% of the participants showed vitamin C depletion/deficiency, 70% were in a prediabetic state, 6% had untreated diabetes. Still, it has been shown that guava fruit consumption might have played a protective role against periodontitis in a malnourished population, regarding the extent and severity of ABL (at least 10% of the participants had a low BMI and were considered as malnourished).
Patil, 2016 [67]	1 year	none	none	none	plasma measurement	A significant decrease in vitamin C was observed in the diabetic periodontitis group as compared with healthy control groups. Type 2 diabetic subjects revealed excessive ROS concentration, therefore more periodontal tissue destruction.
Kunsongkeit, 2019 [64]	2 months	500 mg	tablets	daily for 2 months	plasma measurement	Periodontitis patients with uncontrolled type 2 DM did not have evident benefits by supplementation of 500 mg/day vitamin C.

Abbreviations (in alphabetical order): ABL—alveolar bone loss, AA—ascorbic acid, AAL—ascorbic acid levels, BMI—body mass index, DM—diabetes mellitus, PB—probing depth, PPDs—probing pocket depths, ROS—reactive species of oxygen, SBI—sulcus bleeding index, SRP—scaling and root planning.
